# Sudden death: Neurogenic causes, prediction and prevention

**DOI:** 10.1177/2047487317736827

**Published:** 2017-10-20

**Authors:** Nina Japundžić-Žigon, Olivera Šarenac, Maja Lozić, Marko Vasić, Tatjana Tasić, Dragana Bajić, Vladimir Kanjuh, David Murphy

**Affiliations:** 1 Faculty of Medicine, University of Belgrade, Serbia; 2 Faculty of Technical Sciences, University of Novi Sad, Serbia; 3 Department of Medical Sciences, Serbian Academy of Sciences and Arts, Serbia; 4 School of Clinical Sciences, University of Bristol, UK

**Keywords:** Sudden death, sympathetic, parasympathetic, heart rate variability, baroreflex sensitivity

## Abstract

Sudden death is a major health problem all over the world. The most common causes of sudden death are cardiac but there are also other causes such as neurological conditions (stroke, epileptic attacks and brain trauma), drugs, catecholamine toxicity, etc. A common feature of all these diverse pathologies underlying sudden death is the imbalance of the autonomic nervous system control of the cardiovascular system. This paper reviews different pathologies underlying sudden death with emphasis on the autonomic nervous system contribution, possibilities of early diagnosis and prognosis of sudden death using various clinical markers including autonomic markers (heart rate variability and baroreflex sensitivity), present possibilities of management and promising prevention by electrical neuromodulation.

## Introduction

Sudden death (SUD) is a major health problem all over the world. Estimations are that SUD is responsible for one fifth of all deaths. The most common causes of SUD are cardiac, but there are also non-cardiac reasons, such as neurogenic conditions, catecholamine toxicity, asthma attacks etc.^1^ The diversity of underlying pathology of SUD is the reason why there is neither accurate prediction nor effective management. Yet, a plethora of data suggests that the imbalance of the autonomic nervous system control of the cardiorespiratory system is a common phenomenon linking various pathologies underlying SUD syndrome.

Two components of the autonomic nervous system, the sympathetic and parasympathetic (vagal), act in concert to modulate electrical and mechanical properties of the heart. The balance between the two is fine-tuned by the brain, which processes the inputs from the periphery (baro-reflexes, chemo-reflexes, etc.) and higher brain structures (cortex, limbic system, hypothalamus, etc.). Postganglionic sympathetic nerves that reach the heart release noradrenalin and activate β adrenergic receptors (β1<<β2<β3) to produce the following effects: synthesis of adenylate cyclase, formation of cyclic adenosine monophosphate and activation of protein kinase A. Protein kinase A then phosphorylates structural proteins of ion channels (Na^+^, L type Ca^++^, ultra rapid and slow delayed rectifier K^+^) and key proteins involved in intracellular calcium handling (phospholamban, troponin I, ryanodine type 2 receptors, etc.) endorsing arrhythmogenesis under unfavourable, pathological conditions. On the other hand, vagal nerves that innervate the heart most densely in atria release acetylcholine that binds to muscarinic, M-2 receptors coupled to inhibitory G proteins, activates K^+^ channels and inhibits cyclic adenosine 3′, 5′-monophosphate-dependent stimulation of L type Ca^++^ channels. Cholinergic transmission is further supported by nitric oxide release from cardiac nerves further preventing the occurrence of life-threatening arrhythmias.^1,2^

Heart diseases affect its architecture and the response to neuro-endocrine control mechanisms. They also incite deleterious adaptive remodeling of mechanisms leading to a fatal outcome. Thus the purpose of the present paper is to update knowledge on the neurogenic contribution to the pathogenesis of SUD, the possibilities of early diagnosis/prognosis and present and future directions of prevention. MEDLINE/Pubmed, Web of science, EMBASE and CENTRAL databases were searched for relevant papers on SUD syndrome and the autonomic nervous system.

## Causes of SUD

### Cardiac causes

The vast majority of the cases of SUD occur in the elderly population suffering from cardiac diseases, of which coronary artery disease is the most prevalent. Frequent cardiac diseases associated with SUD also include cardiomyopathies (both hypertrophic and dilated forms) and congestive heart failure.^3,4^ Accumulated evidence suggests that neurogenic dysfunction is central to the development and progression of most cardiovascular diseases including myocardial infarction (MI) and heart failure, and that enhanced sympathetic activity and impaired cardiac parasympathetic responsiveness are negative prognostic indicators for both morbidity and mortality associated with SUD.^5^

During cardiac ischaemia, sympathetic hyperactivity is crucial for the generation of spontaneous ventricular ectopy and life-threatening arrhythmias.^6^ In the past few decades the mechanisms have been elucidated. Cardiac ischaemia has been shown to injure both nerves and myocardial tissue and to elicit denervation and re-innervation of the myocardium, as advocated by sympathetic scintigraphy.^7^ Acute ischaemic necrosis induces sympathetic denervation and the upregulation of β adrenergic receptors at cardiomyocytes inducing receptor hypersensitivity^8^ and hyperresponsiveness to catecholamines. Moreover, in the infarcted area, nerve growth factor (NGF) is released locally in abundance and this triggers increased expression of NGF and of growth associated protein 43 (GAP43). These are transported retrogradely to the left stellate ganglion where they induce nerve sprouting in non-infarcted sites.^9,10^ Uneven sympathetic innervations of the myocardium, and release of neurotransmitters (noradrenalin, neuropeptide Y) in hyper-innervated sites in excess,^11^ induce ionic imbalance and action potential prolongation that increases the risk of arrhythmias and the incidence of SUD.^12^

Ischaemic and other causes of cardiomyopathy, such as infection (usually viral), drug toxicity (mostly antarcycline cytotoxicity), inflammation, autoimmune diseases, and transmissible gene mutations (mostly genes encoding sarcomeric proteins, nuclear envelope and the cytoskeleton) are associated with the formation of scars and fibrosis. Histological studies identified two forms of fibrosis: replacement fibrosis due to cell death and interstitial fibrosis due to collagen accumulation, both of which are governed by the activation of the renin–angiotensin system and β adrenergic system.^13^ Areas of interstitial fibrosis slow or block electrical conduction and its peripheral zone is thought to act as a node for re-entry wave fronts.^14,15^

Regardless of the cause of heart failure (ischaemic, non-ischaemic), the reduction of cardiac output triggers a reflex increase of sympathetic outflow, as well as an increase of renin–angiotensin–aldosterone synthesis and the release of antidiuretic hormone from the hypothalamus, in order to maintain the circulation.^16^ Increased sympathetic activity directed to the failing heart increases heart rate (HR) and causes diastolic calcium leak through ryanodine type 2 (RyR2) receptors. Focally released calcium initiates more calcium release and propagates as a calcium wave that can cause delayed afterdepolarisation and engender premature beats evolving to sustained ventricular tachycardia.^17^ The neuroendocrine arousal in congestive heart failure incites adaptive but harmful mechanisms, NGF downregulation, reduction of sympathetic innervations^18^ and downregulation of β adrenergic receptors.^19^ Loss of adrenergic support is detrimental to the heart inotropism. An alternative scenario was observed in an experimental model of the right ventricular hypertrophy induced by monocrotaline-induced pulmonary hypertension in rats by Kimura and colleagues.^20^ In that model, in spite of the NGF upregulation and sympathetic hyper-innervation, there was a decrease of noradrenalin synthesis and re-uptake suggesting functional denervation occurs due to nerve rejuvenation.^21^

The experimental findings on the role of the sympathetic nervous system in the occurrence of life-threatening arrhythmias is clearly supported by the efficacy of β-blockers in the prevention of SUD in patients suffering from MI, as well as the effectiveness of cardiac sympathetic denervation in patients suffering from ventricular tachycardia and fibrillation storms refractory to drug treatment.^22^

A very small percentage (less than 1%) of the total population affected by SUD are young people, below the age of 35 years.^23^ At autopsy, usually no morphological abnormalities are identified and the underlying cause of SUD remains uncovered. Molecular forensic pathology revealed that the population of young people that succumbed to fatal arrhythmias is affected by hereditary, genetic, disorders caused by mutations in genes encoding structural elements of cardiac ion channels responsible for abnormal electrical activity of the heart.^24^ There is no doubt that the autonomic activity associated with exercise and stress can trigger SUD in individuals suffering from congenital channelopathies, usually presenting normal ECG under baseline physiological conditions. The most common hereditary disorders are congenital long QT syndrome (LQTS), catecholaminergic polymorphic ventricular tachycardia (CPVT) and Brugada syndrome (BrS).

**Figure 1.**
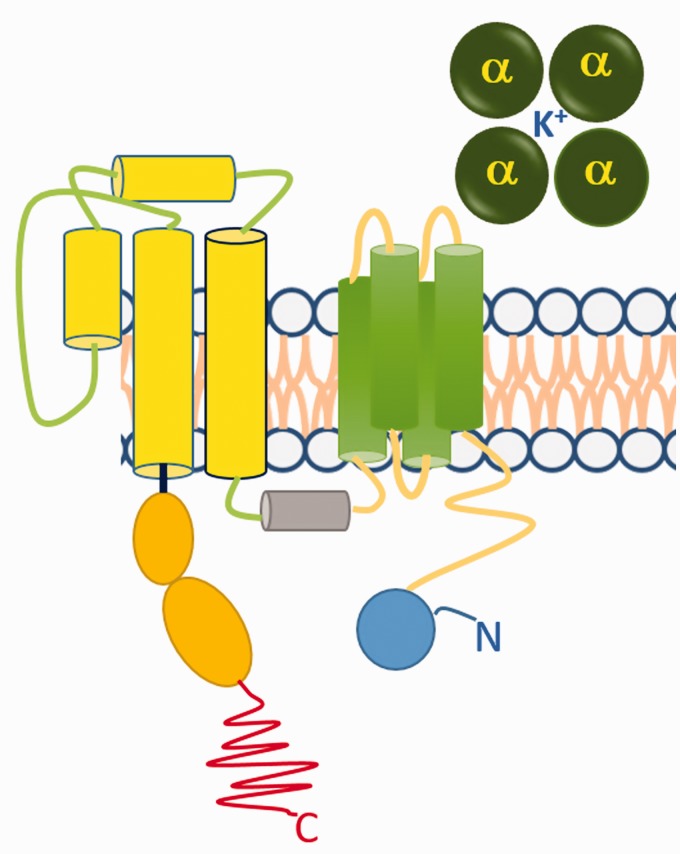
Alpha subunit of the voltage operated K^+^ channel. The voltage operated K^+^ channel is a symmetric tetramer of four alpha subunits around a pore. Each α subunit encoded by the human ether-to-go-go gene (hERG) has six transmembrane helices.

Congenital LQTS is characterised by delayed repolarisation of the myocardium and is genetically a heterogeneous disorder. It is often inherited in an autosomal dominant mode. Hundreds of mutations have been identified in 13 genes including KCNQ1 (LQT1), hERG (LQT2), SCN5A (LQT3), ANK2(LQT4), KCNE1 (LQT5), KCNE2 (LQT6), KCNJ2 (LQT7), CACNA1C (LQT 8), CAV3 (LQT9), SCN4B (LQT10), AKAP9 (LQT11), SNTA1 (LQT12) and GIRK (LQT13).^25^ Among these, the chromosome 7 associated hERG (human ether-à-go-go-related gene, alternative nomenclature KCNH2) mutation was the first reported and is the most prevalent.^26,27^ This gene encodes the constitutive α subunit of voltage-gated K^+^ channels (Figure 1). The α subunit forms the conductance pore of the voltage-gated K^+^ channel and the mutations change modulates its voltage dependence.^28^ To date, nearly 300 different hERG mutations linked to LQT2 have been identified. Such mutations may cause loss of hERG function by one of four main effects: reduced or defective synthesis; defective trafficking from the endoplasmic reticulum to the plasma membrane (resulting in decreased surface expression); defective gating; or defective ion permeation.^29^

The hERG K^+^ channel is responsible for ‘rapid’ delayed rectifier K^+^ current, which plays an important role in ventricular repolarisation. Its malfunction is associated with prolongation of the QRST interval. Long QRST reflects the prolongation of the phase 3 action potential representing the process of cardiac cell repolarisation. As repolarisation becomes prolonged, the left ventricle becomes susceptible to a premature (i.e. early) afterdepolarisation, which triggers rapid polymorphic ventricular tachycardia ‘torsades de pointes’, the phenomenon first described by French physician François Dessertenne in 1960s. The name denotes the twisting of the points of QRS complexes around the isoelectric baseline of the ECG.

CPVT is another heritable arrhythmia syndrome transmissible in autosomal recessive manner, as a result of mutations of genes encoding RyR2 (*CPVT1* 60%) cardiac ryanodine receptor,^30^ or a rare form with mutations of cardiac calsequestrin encoded by the CASQ2 gene (*CPVT2*). Calsequestrin is a protein in the endoplasmatic reticulum that binds calcium and releases it during depolarisation-associated calcium-induced calcium release. Mutations of the *CASQ2* gene disrupts the linear polymerisation of the calsequestrin and reduces its ability to retain calcium inside the reticulum. It is usually diagnosed during exercise or stress by the appearance of ventricular ectopy typically clinically manifested with syncope or SUD.^31^

BrS is in 30% of cases inherited as an autosomal dominant trait cause by loss-of-function mutation of the *SCN5A* gene that encodes cardiac sodium channel (*BrS1*).^24^ At rest, ECG records of the precordial V1–V3 leads show elevation of the ST segment and negative T wave. They have an increased risk of episodes of polymorphic ventricular tachyarrhythmia leading to SUD.

It is noteworthy mentioning that the LQTS and torsades de pointes can be acquired and induced by drugs, both cardiac (class I and class III anti-arrhythmics) and non-cardiac drugs (treatments for widely differing conditions).^32–35^ The mechanism of drug-induced arrhythmias seems to involve direct blockade of the hERG K^+^ channel or inhibition of hERG trafficking.^36^ Knowing this, an in-vitro I_Kr_/hERG assay using the patch clamp technique has been adopted for early screening of drug candidates in a cardiac safety testing programme. ^36,37^ Also, in clinical settings a risk score for predicting QTc prolongation has been proposed and validated.^38,39^

### Neurocardiogenic causes

In 1942, the famous professor of physiology at Harvard Medical School, Walter B. Cannon, published a paper entitled ‘Voodoo Death’ largely known as death from fright*.*^40^ Cannon postulated that, under special circumstances involving social pressure and susceptible psychological profile, death can occur from fright in an otherwise healthy person. He believed that fright can elicit intense activation of the sympatho-adrenal system.^41^ His student Curt Richter challenged his view with evidence that showed that increased vagal tone, without sympatho-adrenergic activation, provoked massive SUD in a colony of rodents whose whiskers were clipped.^42^ It is now established that the sympathetic and parasympathetic part of the autonomic nervous system complements each other in cardiovascular regulation and that life-threatening stressors induce sympathovagal imbalance.^43^

**Figure 2.**
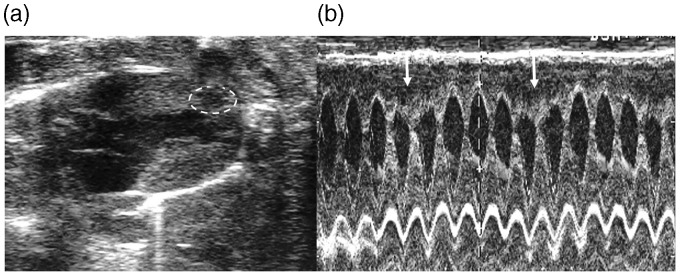
Isoprenaline-induced cardiac dysfunction. (a) Parasternal long axis image at end-systole showing typical akinesia of left ventricular septal wall in one male Wistar rat two hours after 50 mg/kg isoprenaline injection. (b) M mode of long axis view. Dotted circle and arrows indicate akinetic parts of myocardium Source: reproduced with permission of M. Vasić.

In the 1970s, clear experimental evidence that cardiac lesions can be produced as a result of nervous system disease was provided. Hans Selye was the first to produce heart injury in rats by exposing them to stressors and increased concentrations of hormones.^44^ In these animals thrombotic coronary occlusion was not present, and the pattern of myocardial necrosis described by Selye was coagulative myocytolysis whereby myocytes die in a hypercontracted state with early myofibrillar damage and irregular cross-band formation (also termed myofibrillar degeneration and contraction band necrosis). Lesions were multifocal, and the localisation was preponderantly subendocardial, corresponding to the distribution of sympathetic nerve endings. A confirmation for the deleterious effects of excessive adrenergic activation on the heart came from the rat model for stress-induced cardiomyopathy. Infusion of high doses of isoprenaline, a β adrenergic receptor agonist, has been shown to induce injury of the rat heart, pointing to a major role of β adrenergic receptors in catecholaminergic cardiotoxicity.^45^ Figure 2 illustrates akinetic parts of the myocardium of a rat treated with a high dose of isoprenaline.

Decades later, neurocardiogenic heart injuries were reported in humans, giving clinical confirmation to the experimental finding. Acute cardiomyopathy known as broken heart syndrome or Takotsubo cardiomyopathy was first described by Japanese cardiologists^46^ towards the end of the 20th century in the Japanese population. It affected elderly women who experienced great grief. It is clinically indistinguishable from acute coronary syndrome. However, it is not associated with coronary thrombosis and the pathognomonic sign at echocardiography is apical ballooning (akinetic part) of the left ventricle resembling the shape of a ‘tako-tsubo’ ceramic pot used to trap octopus in Japan.^47^ Further clinical evidence for neurocardiogenic injuries came from patients suffering from pheochromocytoma, in which chronic exposure to increased concentrations of circulating catecholamines was evident.^48^ Multifocal subendocardial injuries were also observed in reperfusion injuries during heart transplantation, so-called ‘stoned heart syndrome’, as well as during percutaneous coronary intervention.^49^ In past decades advances have been made in understanding these mechanisms but not yet in providing effective cardioprotection.^50^

Cardiac lesions produced by central nervous system stimulation are morphologically indistinguishable from stress and catecholamine-induced cardiac damage. Back in 1963, Melville and colleagues^51^ produced bradycardia and myocardial necrosis in cats by stimulation of the anterior hypothalamus. Later on, Kannan and collaborators^52^ in 1989 induced an increase in blood pressure (BP) and renal sympathetic outflow in conscious rats by bilateral electrical stimulation of the paraventricular nucleus (PVN) of the hypothalamus or by microinjecting L-glutamate, an excitatory amino acid neurotransmitter, into the PVN. Morphological studies complemented functional studies and provided evidence of direct PVN axonal connections, from the parvocellular part to the respiratory, pre-Bötzinger complex, vasomotor and cardiac neurons in the rostro-ventro-lateral medulla and preganglionic neurons in the inter-medio-lateral column of the spinal cord. The PVN also receives abundant inputs from other part of the hypothalamus, the limbic system and higher brain regions as well as from the periphery.^53^ Such neural network enables the PVN to be a key site in the coordination of behavioural and neuroendocrine response to stress. Furthermore, it has been assumed, both in animals and humans, that the increase of L-glutamate concentrations in the cerebrospinal fluid during cerebral ischaemia stimulates *N*-methyl-d-aspartic acid receptors in PVN and enhances sympathetic drive to the heart^54–57^ suggesting a mechanism of SUD associated with stroke. The PVN is a major source of vasopressin and oxytocin and vasopressin receptors (V) and oxytocin receptors (OT) are found in abundance in the PVN.^58^ Their role is to prime magnocellular neurons and also to integrate the activity of neurons located in the magnocellular and the parvocellular parts of the PVN,^59^ involved respectively in humoral and autonomic neural control of the circulation.^60^ Lozić and colleagues^61,62^ have demonstrated that upregulation of vasopressin V_1A_ and OT receptors in PVN modulates autonomic control of the cardiovascular system and changes the vulnerability of the rat phenotype to stress, thus pointing to possible targets for new drug development in cardioprotection from neurocardiogenic injuries.

In clinical practice arrhythmias are frequently seen in stroke patients without primary heart disease,^63^ and their occurrence correlates to a central increase of sympathetic activity.^64^ Other brain regions affected by stroke have also been associated with sudden autonomic death. Oppenheimer suggested a role for the insular cortex in the pathophysiology of SUD.^65^ It has been reported that right-sided ventromedial prefrontal cortex lesions bear a risk of an exaggerated cardiovascular response.^66^ Myocardial damage and an increase of troponins and Takotsubo syndrome have been reported in humans suffering from epileptic seizures.^67–69^ McMillan and Teasdale^70^ reported a high incidence of sudden death in humans who had mild traumatic brain injury years ago. Clinical evidence of neurocardiogenic damage is usually reported in anterolateral and inferolateral leads of the ECG recording: repolarisation changes in ST segment, peaked T wave and U wave, known as hyperkalemic pattern.^64^

## Markers of SUD

Patients at greatest risk for SUD are those with coronary artery disease and impaired left ventricular function, heart failure secondary to ischaemia or idiopathic dilated cardiomyopathy, hypertrophic cardiomyopathy, documented sustained ventricular tachycardia or fibrillation, and survivors of cardiac arrest. Assessing the cardiovascular function and the degree of impairment of left ventricular ejection fraction (LVEF) is currently used for prognosis in patients with structural heart disease. It is acknowledged that a LVEF of 40% or less is an independent risk factor. However, it should be noted that there is no linear correlation between the degree of left ventricular dysfunction and the prevalence of fatal arrhythmias, the occurrence of which is higher among patients with mid to moderate heart failure, rather than in those with severe failure.^71^ Therefore, multifaceted evaluation using different risk markers increases the accuracy of detecting cardiac risk for life-threatening arrhythmias.^71,72^ Electrocardiographic measures such as standard 12-lead ECG QRS duration, QT dispersion, signal averaged ECG, microvolt-level T wave alternans (a beat-to-beat variability of T wave amplitude that reflects heterogeneity/dispersion of ventricular repolarisation), HR turbulence and HR deceleration capacity, have all been proposed.^73^ In electrophysiological testing, a programmed ventricular stimulation is used for risk stratification. Induction of sustained ventricular tachycardia during programmed ventricular stimulation is a marker of increased risk of recurrent sustained ventricular tachycardia and sudden cardiac death.^74^

**Figure 3.**
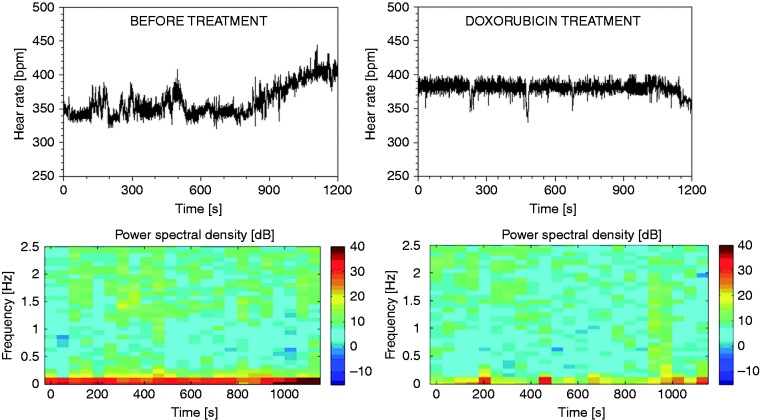
Spectrograms of heart rate variability (HRV) in cardiomyopathy induced by doxorubicin (right). Note the reduction of HRV in lower frequencies (marked in red) by toxic doses of doxorubicin three days before fatal outcome. Source: reproduced with permission of D. Bajić.

**Figure 4.**
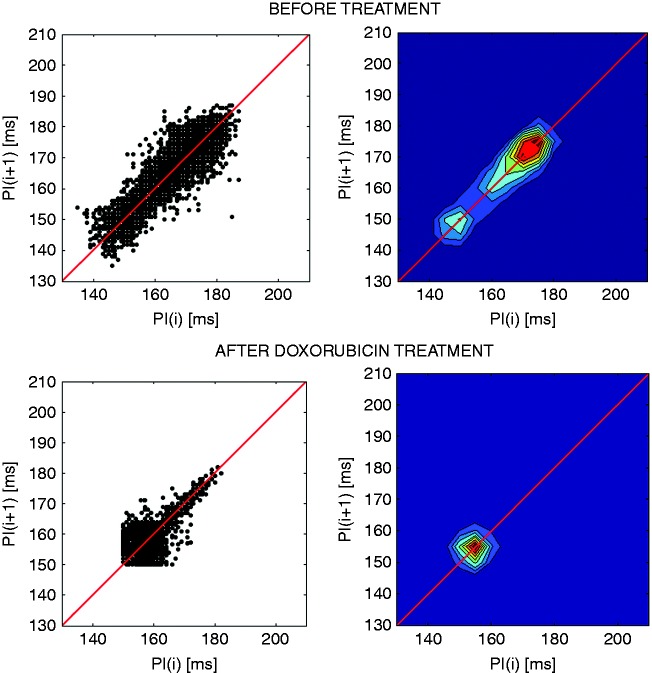
Poincaré plots and the corresponding contour density plots of the heart rate (HR) in one rat before and after treatment with doxorubicin. Note the comet-shaped plot before treatment and the change of the shape after treatment due to a reduction of HR variability three days before fatal outcome. Source: reproduced with permission of D. Bajić.

Among autonomic markers the most appreciated that add to the specificity of LVEF are heart rate variability (HRV) and baroreflex sensitivity (BRS). The clinical relevance of HRV was first observed in 1963 by Hon and Lee,^75^ but it became recognised in the late 1980s when it was confirmed that HRV is a strong and independent predictor of mortality after MI.^76–78^ Many methods in both the time and frequency domain are used to calculate HRV as detailed elsewhere.^79^ However, the true nature of HRV was discovered by the method of spectral analysis in animal models.^80^ In animals and humans, the lower frequencies of the power spectrum of HRV are created by the activity of the sympathetic nervous system directed to the heart, whereas higher frequencies are created by vagal activity modulated by respiration and depict respiratory sine arrhythmia.^80,81^ In elderly individuals, the lower frequency components of the HR spectrum are observed because respiratory sine arrhythmia is lost with ageing.^82^ In diseased individuals, in patients surviving an acute MI and in patients suffering from chronic heart failure or left ventricular dysfunction, reduced or abnormal HRV are indicators of an increased risk of mortality within a few years after an acute MI or after a diagnosis of congestive heart failure or left ventricular dysfunction.^5,83–85^ HRV has proved to be useful in predicting SUD in patients suffering from non-cardiac causes.^69,86^ Picturesque methods for the assessment of HRV are colour spectrograms (Figure 3) and Poincaré plots (Figure 4). When the non-linearity of the HR signal is increased, the reliability of linear methods is reduced, and additional insight is provided using non-linear parameters. Complexity-based analysis of fractal dimension and approximate entropy of HRV increase the accuracy of risk stratification. They were also proved to be useful in predicting the risk in Takotsubo cardiomyopathy.^87^ HRV and BP variability have been used for the assessment of preclinical drug safety for drug-induced cardiotoxicity.^88–90^

The baroreflex is the main negative feedback corrector of arterial BP, and evaluation of BRS is an established tool for the assessment of autonomic control of the cardiovascular system.^91^ Baroreceptors located in the upper part of the body, aortic arch and carotid sinuses sense subtle changes in central BP and adjust circulation to all organs, but especially to the brain, on a beat-to-beat basis. The more sensitive the baroreflex, the less variable is the BP, and the lower is the risk of stroke.^92^ Under physiological conditions, BRS decreases during exercise and stress,^93,94^ in order to permit concomitant increase of arterial BP and HR to supply adequate circulation to the body. However, a decrease in BRS occurs in disease, in survivors of MI and in patients suffering from chronic heart failure, and represents an independent risk factor for SUD.^5,91^

BRS can be assessed in an old fashioned manner using pharmacological tools (Oxford method) and using novel methods from spontaneously occurring BP and pulse interval variability in the low frequency and high frequency spectral bands. The Oxford method requires injections of short-acting vasoconstrictor or vasodilating drugs to trigger a reflex HR response, but this carries serious risks for the patient and is thus not widely used in clinical practice. However, the sequence method assesses the BRS from spontaneously and concomitantly occurring systolic BP and pulse interval increasing/decreasing values,^95^ thus circumvents the use of drugs and is without risk for patients, so may be applicable in wide populations. Several useful parameters have been proposed to evaluate the function of the spontaneous baroreflex. Experimental evidence indicates that an increase in spontaneous BRS over an increase in operating range indicates allostatic overload and may precede the exhaustion of the neurogenic mechanisms.^94^

## Possibilities of SUD prevention

The current management of SUD involves drugs and electrical devices for correction of arrhythmias. In patients with ischaemic heart disease and congestive heart failure β-blockers were found to be most effective and reduced total mortality. Other anti-arrhytmic drugs are not well tolerated because they have pro-arrhythmogenic action. Class I anti-arrhythmic drugs in patients with ischaemic heart disease, especially class 1C, have been shown to increase mortality. Class III, dofetilide and amiodarone have no effect on mortality while *d*-sotalol increases mortality.^96^ A new drug, dronedarone, is promising.^97^ β-Blockers have been applied with success in women suffering from hereditary long QT syndrome, in the postpartum period when the risk from cardiac events is several fold increased.^98^ In the therapy of coronary artery disease and congestive heart failure the so-called non-anti-arrhythmic drugs are useful in the prevention of SUD, although their effect is indirect and delayed. These include angiotensin-converting enzyme inhibitors and angiotensin II AT1 receptor blockers, statins, and nutrients such as fish oil.^98^

In patients with very low LVEF, asymptomatic ventricular arrhythmias and inducible ventricular tachycardia/fibrillation, an implantable cardioverter-defibrillator (ICD) is superior to conventional drug therapy. In secondary prevention of SUD, in patients with sustained ventricular tachycardia/fibrillation or survivors of sudden cardiac death drug treatment alone is ineffective and can be used as an adjunct to ICD to decrease episodes of ventricular tachycardia/fibrillation and non-sustained ventricular tachycardia to reduce ICD discharge, to slow the rate of ventricular tachycardia and to increase haemodynamic stability.^96,99^

Patients with advanced heart failure and low LVEF in which symptoms progress despite optimal pharmacotherapy and ICD device implantation may benefit in future from devices engineered to restore sympathovagal balance to the heart by electrical neuromodulation. Several options have been investigated in animal models, involving baroreflex activation therapy (BAT) and vagal stimulation. In dogs with experimentally induced heart failure and very low LVEF, BAT reduced sympathetic tone and enhanced vagal tone, reduced susceptibility to life-threatening arrhythmias and prolonged survival.^100^ The first-into-man study, although performed on a small sample and with a short follow-up period confirmed the beneficial effects of BAT in advanced stages of chronic heart failure.^101^ In the first randomised and controlled trial patients with heart failure with reduced ejection fraction also benefited from BAT treatment.^102^ An alternative approach to restore the sympathovagal balance to the heart is to stimulate the vagus nerve directly. Experimental studies indicate that several mechanisms are likely to be involved in the beneficial effect of vagal stimulation.^103^ These include anti-adrenergic effects, anti-apoptotic effects, an increase of nitric oxide synthesis and anti-inflammatory effects.^104^ The first-in-man study with chronic heart failure confirmed that the method is safe and well tolerated, and significant improvement of the LVEF and life quality was observed.^105^ There are experimental indices in rabbit and rats that BRS may be increased by chronic exposure of baroreceptors to a medium intensity static magnetic field.^106^ Studies indicate that a medium intensity static magnetic field affects BRS and cardiovascular haemodynamics by modulating microcirculation, neurotransmission and the rate of biochemical reactions.^107^

Finally, it can be concluded that in spite of the development of new anti-arrhythmic drugs and DCI devices, SUD remains a frequent cause of death worldwide. In the past few decades progress has been made in the multifaceted risk stratification approach by the introduction of autonomic markers into clinical practice. A new approach of treatment by electrical neuromodulation is under development for the prevention of SUD in advanced stages of congestive heart failure. The results of large scale clinical studies are still awaited to confirm the clinical efficacy and tolerability of electrical neuromodulation that may hopefully become a new cornerstone in the prevention of SUD in these patients.
